# Alcohol Consumption Practices among Married Women of Reproductive Age in Nepal: A Population Based Household Survey

**DOI:** 10.1371/journal.pone.0152535

**Published:** 2016-04-01

**Authors:** Narbada Thapa, Krishna Kumar Aryal, Rupendra Puri, Saraswoti Shrestha, Sheela Shrestha, Pukar Thapa, Suresh Mehata, Pushpa Thapa, Megha Raj Banjara, Babill Stray-Pedersen

**Affiliations:** 1 Nepalese Army Institute of Health Sciences, Kathmandu, Nepal; 2 Institute of Clinical Medicine, University of Oslo, Oslo, Norway; 3 Nepal Health Research Council (NHRC), Government of Nepal, Kathmandu, Nepal; 4 District Ayurveda Health Centre, Department of Ayurveda, Ministry of Health and Population, Government of Nepal, Kathmandu, Nepal; 5 Initiative for Research Education and Community Health—Nepal (InREACH Nepal), Kathmandu, Nepal; 6 National Health Education, Information and Communication Centre, Ministry of Health and Population, Government of Nepal, Kathmandu, Nepal; 7 Kathmandu Medical College, Kathmandu, Nepal; 8 Ipas Nepal, Kathmandu, Nepal; 9 Central Department of Microbiology, Tribhuvan University, Kathmandu, Nepal; 10 Division of Women and Children, Rikshospitalet, Oslo University Hospital, Oslo, Norway; Hospital Universitario LA FE, SPAIN

## Abstract

**Background:**

Alcohol chemically known as ethanol, causes several health, economic and social consequences across the world. Literatures suggest potential harm of alcohol drinking by pregnant women especially to the fetus and the mother. Despite anumber of significant public health problems related to alcohol consumption, this area has been ignored in Nepal and information at the national level is limited. Thus this study aimed at finding the prevalence of alcohol consumption among married women of reproductive age.

**Methods:**

A nationally representative household survey was carried out from April to August 2013 by taking 16 districts across all 15 eco administrative regions. From the selected districts, 86 village development committees and 14 municipalities were selected as primary sampling units using probability proportionate to size, followed by random selection of 3 wards from each primary sampling unit. Finally, 30 households within each ward were selected using systematic random sampling, and one married women of reproductive age from each household. A total of 9000 married women of reproductive age were interviewed using a semi-structured questionnaire, on alcohol consumption practices including environmental factors and socio demographic characteristics and were included in the analysis.

**Results:**

National prevalence of alcohol consumption ever among married women of reproductive age was 24.7% (95% CI:21.7–28.0), last 12 months 17.9% (95% CI:15.3–20.7) and last 30 days (current drinking) 11.8% (95% CI:9.8–14.1). There was substantial variation among the districts ranging from 2% to 60%. Multivariable analysis suggests women with no education or within formal education, dalit and janajatis ethnicity, whose husbands drink alcohol, who brew alcohol at home and women from mountains were significantly at higher risk of consuming alcohol. Among the women who drank alcohol in last 12 months, a substantial proportion of them drank home brewed alcoholic beverages (95.9%, 95% CI:94.3–97.4).

**Conclusion:**

Alcohol consumption was common practice among married women of reproductive age in Nepal with variation among the subgroups of population. Thus, further investigation and behavior change communication interventions to reduce alcohol consumption especially among the women with higher risk of drinking is essential.

## Introduction

Alcohol consumption among reproductive age women has become a significant public health problem worldwide and it varies by country and region. Drinking during pregnancy has detrimental effects to the fetus. A substantial proportion of pregnant women and women of child bearing age in developed countries [1,2,3]consume alcohol despite being clearly established as a teratogen since the 19th century. Pre-pregnancy alcohol consumption has been consistently identified as predictors of prenatal alcohol use[4].

Though there are reports suggesting more women than men are lifetime abstainers of alcohol globally[5], substantial proportion of women in Nepal consume alcohol[6,7,8]. Alcohol has been socially and culturally acceptable among many ethnic groups such as Janajatis in Nepal. Population groups who do not fall to these ethnicity also have been found to be increasingly consuming alcohol[9]. WHO STEPS surveys show that the current drinking by women aged 15–69 years in Nepal appears to be decreasing; 16.1% in 2007/08and 7.1% in 2012/13, but proportion with harmful use of alcohol among the currently drinking women have risen in those 5 years[6,8].

Despite enormous public health problems related to alcohol consumption, information on national level is still lacking in Nepal. Thus present study intended to provide scientific evidence on alcohol consumption practices among married women of reproductive age (MWRA) in Nepal which add knowledge in a novice context and at the same time it may help national policies and plans in this area.

## Materials and Methods

A nationally representative cross-sectional survey was carried out from April to August 2013 to determine the prevalence of alcohol consumption practice among MWRA. Of 75 districts in the country, 16 were selected representing three ecological belts (mountains, hills and the Terai) and the five administrative regions of Nepal. A total of 9,000 MWRA residing in the study area were interviewed on alcohol consumption practices and reproductive health history.Sample size was calculated to allow a maximum of 16 sub group analysis.

As Nepal is divided by three ecological belts (geographical division based on altitude) and five development regions (administrative division) into 15 sub-regions (3X5 = 15), for current study one district was randomly selected from each of these 15 sub-regions[10]. In addition, one more district from the hills in the central region was purposively added to the sample considering the greater number of districts in the hills compared to the mountains and the Terai. Mountains, hills and the Terai are three ecological belts as described in the variables section below. The village development committees (VDCs) and municipalities of the 16 selected districts made the primary sampling units (PSUs) and were listed in alphabetical order separately for the three strata (ecological belts). A total of 100 PSUs were selected from each of these strata using probability proportionate to size (PPS) sampling method based on national population proportion as per the housing and population census 2011 of Nepal [11] which led to the selection of 50 PSUs from the Terai, 33 from hills and 17 from mountains. Among 100 PSUs, 86 were VDCs and 14 were municipalities. VDC and municipality are administrative structures below the district level and wards are the lowest administrative unit in Nepalese administrative structure and each VDC is composed of 9 wards, whereas number of wards in each municipality ranges from 9 to 35.

From the PSU, three wards were selected as secondary sampling unit (SSU) by simple random technique. In the final step, 30 households were selected from each SSU using systematic random sampling and one eligible MWRA from each household was recruited as study sample. In case of unavailability of eligible candidates in sampled household adjoining household was sought. We had a cent percent response in the survey.

### Data Collection Procedure

A household interview was conducted among 9000 MWRA using a pre-tested semi structured questionnaire in Nepali language. Interviewers and supervisors with health background from the same regions were recruited and trained on sampling techniques and interview schedule. Information on socio-demographic characteristics (age, ethnicity, religious status, educational status, occupational status, etc) alcohol consumption practices and environmental factors related to it (home brewing, husband's drinking status, etc) were obtained. For the alcohol consumption related questionnaire, major sections was adopted from the GENACIS questionnaire version November 2001 of University of North Dakota, School of Medicine and Health Sciences [12] and WHO NCD STEPS instrument version 2.2[13].

### Major Variables in the Study

#### Level of education

Education level was defined based on the literacy status. No education referred to those, who could not read and write. Women with informal education were those who could read and write but without any formal education such as taken some adult education classes. Other levels were categorized as per the number of school years: primary education (5 years of schooling completed), secondary education (10 years of schooling completed), higher (more than 10 years of schooling completed).

#### Ethnicity

In Nepal the commonly used ethnic classification has 6 categories: 1) Dalit (marginalized group of population relatively with lower socio economic and education status), 2) Disadvantaged Janajatis (Disadvantaged group of people and also indigenous group of population relatively with lower socio economic and education status), 3) Disadvantaged non Dalit Terai Caste Groups (Disadvantaged group of people from the Terai, the lowlands, relatively with lower socio economic and education status but not the dalit groups), 4) Religious Minorities (Muslim, Christian etc), 5) Relatively advantaged Janajatis (Indigenous group of people however with relatively higher socio economic status such as Newar, Thakali, Gurung) &6) Upper Caste Groups (Population with relatively higher socio economic and education status and mostly Brahmins, Chhetris, Thakuri).

#### Type of family

We recorded type of family in three categories. Nuclear meant only parents and children, joint meant grandparents, parents and children while extended meant including families of brothers as well as grandparents and children.

#### Place of residence

Based on the central bureau of statistics, we categorized VDCs as rural and municipalities as urban in this study.

#### Ecological belt

Nepal is geographically divided into three belts north to south based on altitude. Mountains, hills and the Terai are three ecological belts where mountains refers to the high mountain region, hills refers to adjacent region in a lower altitudinal belt while the Terai refers to the lowest terrain in the country ranging from 70 to 700 meters above the sea level.

#### Alcohol consumption

Alcohol consumption in this study meant consumption of at least one alcoholic drink and based on the time we have considered three different variables for this. Alcohol consumption ever meant consumption of at least one alcoholic drink ever in their lifetime, alcohol consumption in last 12 months meant consumption of at least one alcoholic drink in the last 12 months. Alcohol consumption in last 30 days meant consumption at least one alcoholic drink in the last 30 days. Last 30 days consumption has been termed as current drinking and has been used interchangeably in this manuscript.

This study fully complied with the ethical guidelines of the Nepal Health Research Council (NHRC). It was approved (Ref. No. 554; Ethical Approval: 12 Nov. 2012) by the national Ethical Review Board (ERB) at the NHRC. Formal permission was taken from the concerned authorities in the selected districts, VDCs and municipalities. An informed written consent was obtained from all the participants before the interview.

### Data analysis

Data entry, data cleaning and normalization were done in Epi-Info version 3.5.1 and STATA 12.0 SE version was used for the analysis. Complex sample analysis was adopted for finding out the prevalence, which also accounted for cluster effect and weighting of the data to ensure representation of the entire target population of the country. The three ecological belts were considered the strata and VDCs were considered PSU for the complex sample analysis. All estimates were weighted by sample weights, and are presented with 95% Confidence Intervals (CIs).

Sample weights were calculated considering the multiple stages of sampling in which probability of selection was obtained for each level of sampling starting from the selection of districts at the first level up to the selection of individual household. There were 4 levels of sampling until the household selection and thus 4 probabilities were multiplied to get individual probability for 9000 sample. The reverse of the final probability was used as the individual weight and was used in the analysis.

In addition to the complex sample analysis for the prevalence of the alcohol consumption, we also did a bi-variate analysis resulting in crude odds ratio and multivariate analysis resulting in adjusted odds ratio. Multivariate analysis used the socio-demographic and other environmental variables to calculate the effects of individual independent variables.

## Results

Information from 9000 MWRA’s interviews (100% response rate) in 16 districts of Nepal were included in the current analysis. The alcohol consumption was analyzed in three categories—ever consumed, consumed in last 12 months and consumed in last 30 days (current drinking) as described in methods section.

### Socio-demographic characteristic of the respondents and alcohol consumption

#### Socio-demographic Characteristics

Almost half (47%) of the MWRA were in the age group 20–29 years,31% were 30–39 years, 15% were 40–49 years and 6.9% were 15–19 years of age. One fifth (20.3%) of them had no education, 22.5% had informal education,13.5%, 34.1% and 9.5% had primary, secondary and higher education, respectively. Higher proportion of the MWRA were from Janajati ethnic (42.1%), followed by upper caste ethnic (39.4%), disadvantaged ethnic (11.9% from Dalit and 5.1% from Non-dalit terai caste). 1.5% of them were from religious minority group such as Muslim. Ethnic classification for this study is described in the materials and methods section. However, in this study both Advantaged and Disadvantaged Janajatis were grouped into a single category of Janajatis owing to the similar culture with regards to the alcohol consumption. More than half (56.6%) of them were living in nuclear family, 37.6% in a joint family and 5.8% in an extended family. Of the 9000 MWRAs, 42.8% of them had husband who ever drink alcohol and 23% brew alcohol at home "Table 1".

**Table 1:** Prevalence of alcohol consumption among MWRA by socio-demographic characteristics.

	N (un-weighted)	Ever Consumed		Consumed in last 12 months		Consumed in Last 30 Days	
		Weighted %	95% CI	Weighted %	95% CI	Weighted %	95% CI
**Age (Years)**							
15–19	618	21.7	15.7–29.2	13.8	10.3–18.4	9.9	7.1–13.8
20–29	4232	21.6	19.2–24.3	14.6	12.5–16.9	9.1	7.5–11.0
30–39	2797	26.5	22.6–30.8	20.1	16.6–24.2	13.6	10.6–17.2
40–49	1353	31.6	26.4–37.2	25.0	20.9–29.5	17.0	13.9–20.5
**Level of education**							
No education	1826	25.4	20.7–30.8	20.5	15.7–26.3	17.0	12.6–22.5
Informal	2026	30.5	25.2–36.4	22.7	18.8–27.1	15.8	12.7–19.4
Primary	1217	27.2	23.1–31.7	20.0	16.4–24.3	14.0	10.9–17.7
Secondary	3072	21.3	18.5–24.5	14.4	12.0–17.2	7.9	6.5–9.7
Higher	859	20.0	15.0–26.2	12.6	8.5–18.3	5.2	3.8–7.1
**Ethnicity**							
Dalit	1067	21.8	17.6–26.5	15.2	11.6–19.6	9.6	7.1–12.9
Janajati	3791	38.2	33.7–42.8	29.0	24.6–33.9	20.8	17.0–25.1
Disadvantaged non-dalitterai caste	461	8.6	5.7–12.7	6.5	3.9–10.8	4.2	2.7–6.6
Religious minorities	135	3.3	1.2–8.5	2.9	1.1.-7.5	2.2	0.8–6.4
Upper caste	3546	12.7	11.1–14.1	7.6	6.4–9.0	3.2	2.6–4.0
**Drinking status of husband**							
Yes	3849	35.2	31.0–39.6	27.4	23.7–31.5	20.7	17.4–24.4
No	5151	17.2	14.9–19.8	11.0	9.1–13.3	5.4	4.3–6.9
**Type of family**							
Nuclear	5094	26.1	22.5–30.1	18.9	16.1–22.1	12.5	10.4–15.0
Joint	3380	22.4	19.6–25.5	16.1	13.3–19.3	10.7	8.6–13.2
Extended	526	25.6	20.2–31.9	19.2	13.7–26.2	11.9	7.6–18.2
**Home brewing**							
Yes	2085	51.3	46.4–56.2	45.4	39.6–51.4-	36.1	30.7–41.7
No	6915	15.9	13.9–18.2	8.8	7.5–10.3	3.8	3.1–4.7
**Total Respondents**	**9000**	**24.7**	**21.7–28.0**	**17.9**	**15.3–20.7**	**11.8**	**9.8–14.1**

#### Alcohol consumption

Of 9000 study population, one quarter (24.7%, 95% CI: 21.7–28.0) of them were found to have consumed alcoholic beverages in their lifetime and 17.9% (95% CI: 15.3–20.7) in last 12 months. Alcohol consumption was higher in increased age, however, the difference in ever consumption is statistically significant only between 20–29 years age group (21.6%, 95% CI: 19.2–24.3) and 40–49 years age group (31.6%, 95% CI: 24.6–37.2).

MWRAs with no or informal education were more likely to consume alcohol (ever) than those with the higher education. Women from Dalit and Janajati ethnic group ever consumed alcoholic beverages more than other caste groups. Similarly, MWRAs who belong to Dalit and Janajati ethnic, had husbands who drink, who brew alcohol at home were more likely to consume alcohol than those of other women in the study. There were non-significant differences in alcohol consumption by family type “Table 1”.

Similar to ever consumption, the prevalence of alcohol consumption in last 12 months was found to be higher among the MWRA with informal education and ethnic group; Janajatis and Dalit. Almost one third (29%, 95% CI: 24.6–33.9) of MWRA from Janajati ethnic groups consumed alcohol in last 12 months. Women whose husband drank alcohol had higher (27.4%, 95% CI: 23.7–31.5) prevalence of last 12 months alcohol consumption compared to those (11.0%, 95% CI: 9.1–13.3) whose husbands did not drink.

One forth (23%) of family brewed alcoholic beverages at home and consumption was higher among the women who brew alcohol at home than those not brewing alcohol at home, "Table 1".

#### Types of alcohol consumed

Among those who consumed alcoholic beverages in the last 12 months, home-brewed alcohol was the most commonly consumed type of alcohol for a huge majority (95.9%, 95% CI: 94.3–97.4) of the women. Ethanol concentration in these home-brewed alcohol from the same project has been presented elsewhere[14]. Of those women, 7.1% (95% CI: 4.8–9.4) consumed commercially available alcoholic beverages such as beer, whiskey, wine and about 3.0% (95% CI: 1.8–4.3) consumed both types of alcoholic beverages.

#### Current drinking prevalence

The overall prevalence of current alcohol drinking among MWRA was 11.8% (95% CI: 9.8–14.1). MWRA more likely to consume alcohol (have drunk alcohol during the last 30 days) if they were of Janajati ethnic origin, 40–49 years of age, did not have more than primary education, brewed alcohol at home, had husbands who drank alcohol than MWRA without these characteristics. The consumption was 20.8% (95% CI: 17.0–25.1) and 20.7% (95% CI: 17.4–24.4)among MWRA of Janajati ethnic and had husbands who drank alcohol, respectively “Table 1”.

#### Alcohol consumption by ecological belts, place of residence and districts

MWRA from mountains were more likely to drink alcohol than those from the Terai and hills. The ever drinking prevalence among MWRA residing in mountains was 33.8% (95% CI: 27.4–40.7) and the current drinking prevalence was16.1% (95% CI:12.8–20.1). By residence in terms of urban and rural, prevalence of alcohol consumption appeared similar "Table 2".

**Table 2:** Prevalence of alcohol consumption among MWRA by ecological belt and place of residence.

	N (%)Un-weighted	Ever Consumed		Consumed in last 12 months		Consumed in Last 30 Days	
		Weighted %	95% CI	Weighted %	95% CI	Weighted %	95% CI
**Total respondents**	**9000**	**24.7**	**21.7–28.0**	**17.9**	**15.3–20.7**	**11.8**	**9.8–14.1**
**Ecological belts**							
Mountain	1530	33.8	27.4–40.7	20.5	16.0–25.9	16.1	12.8–20.1
Hill	2970	30.1	24.7–36.2	23.7	19.3–28.8	15.0	11.6–19.2
Terai	4500	17.9	15.1–21.1	11.6	9.7–13.8	7.9	6.5–9.7
**Place of residence**							
Rural	7740	24.7	21.7–28.0	17.3	14.7–20.2	11.9	9.9–14.3
Urban	1260	24.5	16.9–34.1	21.1	14.6–29.4	11.0	7.5–15.9

CI: Confidence Interval.

**Fig 1:**
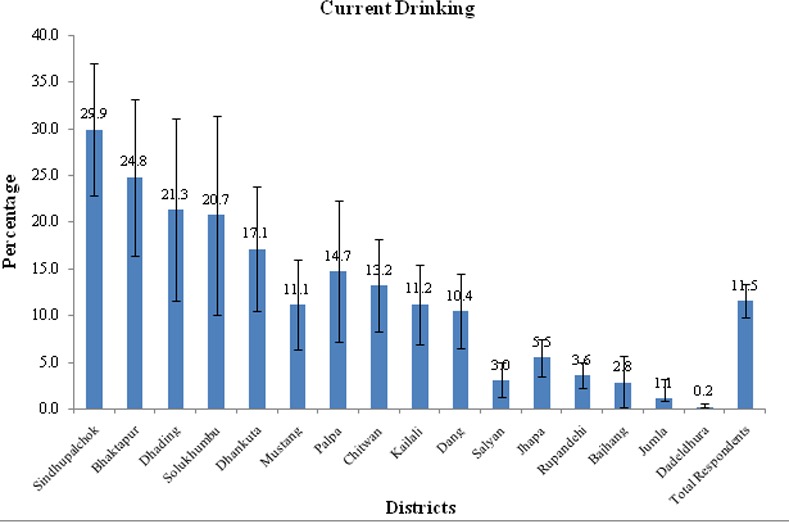
Prevalence of current drinking among MWRA by districts.

Of the 16 districts included in the study, the prevalence of ever alcohol consumption varied from 2% in the western districts of Nepal to 60% in the districts of central Nepal. Among them the highest prevalence of current drinking was found in Sindhupalchowk district (30%, 95% CI: 22.8–37.0), followed by Bhaktapur (25%, 95% CI: 16.4–33.2), Dhading (21%, 95% CI: 11.5–31.1) and Solukhumbu district (21%, 95% CI: 10.0–31.4), respectively "Fig 1".

### Socio demographic correlates of alcohol consumption by MWRA

Table 3 demonstrates the crude and adjusted odds ratios for association between alcohol consumption among MWRA and covariates. Alcohol consumption was found to have been affected by level of education, caste/ethnicity, drinking status of husband, residence in terms of mountains, hills and the Terai, and home brewing of alcoholic beverages.

**Table 3:** Crude and adjusted odds ratio for correlates of alcohol consumption among MWRA.

	Ever Consumed		Consumed in last 12 months		Consumed in Last 30 Days	
	Crude OR (95%CI)	Adjusted OR (95% CI)	Crude OR (95%CI)	Adjusted OR (95% CI)	Crude OR (95%CI)	Adjusted OR (95% CI)
**Age (Years)**						
15–19	1	1	1	1	1	1
20–29	0.99 (0.70–1.41)	0.90 (0.63–1.28)	1.06 (0.77–1.45)	0.93 (0.65–1.34)	0.91 (0.61–1.35)	0.71 (0.45–1.10)
30–39	1.29 (0.89–1.88)	1.11 (0.75–1.61)	1.56 (1.07–2.29)	1.27 (0.89–1.83)	1.42 (0.97–2.08)	0.93 (0.59–1.45)
40–49	1.66 (1.16–2.37)	1.31 (0.79–2.13)	2.07 (1.40–3.05)	1.46 (0.98–2.16)	1.85 (1.24–2.74)	0.94 (0.55–1.60)
**Level of education**						
No education	1	1	1	1	1	1
Informal	1.29 (1.01–1.63)	1.25 (0.96–1.64)	1.13 (0.84–1.51)	1.06 (0.83–1.36)	0.91 (0.64–1.30)	0.81 (0.61–1.07)
Primary	1.09 (0.82–1.46)	**1.31 (1.03–1.68)**	0.96 (0.71–1.32)	1.20 (0.94–1.53)	0.79 (0.55–1.14)	0.93 (0.69–1.25)
Secondary	0.79 (0.60–1.04)	1.07 (0.84–1.39)	0.65 (0.48–0.88)	0.89 (0.71–1.14)	0.42 (0.29–0.60)	**0.53 (0.41–0.71)**
Higher	0.73 (0.47–1.14)	1.25 (0.85–1.82)	0.55 (0.32–0.96)	0.96 (0.66–1.41)	0.27 (0.17–0.40)	**0.46 (0.29–0.72)**
**Ethnicity**						
Dalit	1	1	1	1	1	1
Janajati	2.22 (1.80–2.73)	**1.72 (1.42–2.09)**	2.29 (1.62–3.22)	**1.52 (1.14–2.03)**	2.47 (1.69–3.61)	**1.61 (1.17–2.20)**
Disadvantaged non-dalit terai caste	0.33 (0.21–0.52)	**0.53 (0.35–0.82)**	0.39 (0.22–0.68)	0.70 (0.41–1.19)	0.41 (0.24–0.72)	0.76 (0.41–1.41)
Religious minorities	0.12 (0.04–0.35)	**0.26(0.09–0.77)**	0.17 (0.06–0.46)	0.43 (0.16–1.20)	0.21 (0.06–0.67)	0.73 (0.24–2.22)
Upper caste	0.52 (0.41–0.65)	**0.64 (0.54–0.76)**	0.46 (0.31–0.66)	**0.62 (0.47–0.83)**	0.31 (0.22–0.43)	**0.56 (0.43–0.72)**
**Drinking status of husband**						
No	1	1	1	1	1	1
Yes	2.61 (2.13–3.12)	**1.89 (1.59–2.25)**	3.04 (2.43–3.81)	**2.13(1.75–2.61)**	4.53 (3.63–5.63)	**3.06 (2.46–3.81)**
**Type of family**						
Nuclear	1	1	1	1	1	1
Joint	0.81 (0.71–0.93)	0.84 (0.71–0.99)	0.82 (0.68–0.98)	0.88 (0.72–1.06)	0.83 (0.70–0.99)	0.93 (0.75–1.16)
Extended	0.97 (0.69–1.38)	1.11(0.84–1.43)	1.02 (0.70–1.47)	1.20 (0.85–1.71)	0.94 (0.59–1.51)	1.16 (0.78–1.73)
**Place of residence**						
Rural	1	1	1	1	1	1
Urban	0.98 (0.61–1.58)	1.12 (0.66–1.89)	0.78 (0.49–1.24)	1.72 (0.99–2.98)	0.91 (0.60–1.38)	1.31 (0.91–1.90)
**Ecological belt**						
Mountain	1	1	1	1	1	1
Hill	0.84 (0.56–1.26)	0.70 (0.42–1.19)	1.20 (0.80–1.80)	1.01 (0.62–1.68)	0.91 (0.61–1.37)	0.76 (0.51–1.17)
Terai	0.42 (0.29–0.61)	**0.41 (0.24–0.72)**	0.50 (0.35–0.73)	**0.51 (0.32–0.80)**	0.44 (0.31–0.63)	**0.47 (0.32–0.69)**
**Home brewing of alcohol**						
No	1	1	1	1	1	1
Yes	5.57 (4.23–7.31)	**3.72 (2.96–4.69)**	8.65 (6.41–11.66)	**6.03 (4.69–7.76)**	14.27 (10.72–19.0)	**9.21 (7.04–12.06)**

MWRAs with higher education were two times less likely to be current drinkers than those with no education. There was a significant difference in the likelihood of alcohol consumption (ever, last 12 months and current) between ethnic groups even after adjusting the covariates. Compared to the Dalit ethnic, MWRAs from the Janajati ethnic group were almost two times more likely to drink alcohol (adjusted OR = 1.61, 95% CI: 1.17–2.20), and in contrast the upper caste ethnic group women were almost two times less likely to consume alcohol (adjusted OR = 0.56, 95% CI: 0.43–0.72) as current drinkers.

Those MWRAs whose husbands drank alcohol, who brewed alcohol at home and were from mountains were more likely to consume alcohol. Women with husbands who drank were three times more likely to drink than those who did not have husbands who drank (adjusted OR = 3.06, 95% CI: 2.46–3.81). Similarly, the consumption in the last 30 days was nine times higher among women who brewed alcohol at home (9.21, 95% CI: 7.04–12.06) than those who did not brew alcohol. Women from mountains were more than twice as likely current drinkers than those from Terai. Factors such as age, type of family, residence in terms of urban and rural were not found to have an independent effect on alcohol consumption “Table 3”.

## Discussion

Having an insight into MWRA's alcohol consumption practice and pattern would be significant not only in terms of alcohol use reduction intervention, but also with the future implication of NCD prevention, maternal and newborn health promotion. This paper comes as an endeavor to highlight MWRA's alcohol consumption practice, together with socio-demographic correlates of alcohol use. We believe that the robust study design and large sample size render the prevalence findings, overall, 12% of MWRAs had consumed alcohol in the last 30 days, 18% in the last 12 months and 25% some time during their life. The evidence may be a credible and solid basis for targeted alcohol-preventive interventions among MWRAs in Nepal.

Despite being quite an uncommon phenomenon among the women, literature reveals increasing trend of alcohol consumption among the women[15,16]. Eighteen percent drinking alcohol in the last 12 months in current study is comparatively higher than those of Indian study findings of 5.8%[17] and 2.8%[18]women drinkers, respectively. Similarly, the current drinking prevalence was slightly higher than that of the NCD STEPS survey in Nepal (7.1%)[6]. The difference could be due to the difference in sample population in which the STEPS survey takes any adult women aged 15–69 years while the current study had considered MWRA.

Having looked over various socio-demographic strata, the alcohol ever use, alcohol consumption in the last 12 months, and the last 30 days remained higher if MWRAs were of Janajati ethnic origin, 40–49 years of age, did not have more than primary education,lived in mountains, had husbands who drank alcohol and brewed alcohol at home. These findings indicate the groups at risk of alcohol-related harms and potentially require tailored interventions.

Of them, one to pay harder is 40–49 years age group, as with the advancement in age, they are likely to have higher risk of non communicable diseases. The fact that the significant number of drinkers is from the older age group indicates the rising burden of chronic disease, as the older age population (45–69 years) in Nepal also has significantly higher rates of major NCD risk factors: current smoking, physical inactivity, overweight, raised blood pressure, raised blood sugar and raised total cholesterol [19].

With reference to 15–19 years, the likelihood of consuming alcohol in the last 12 months was 1.68 [95% CI: 1.03–2.73] times higher among 40–49 years. This finding was in concordance with a recent study in Nepal [20]that found women above 50 years of age to have twice the risk of being a current drinkers than younger women[20]. Increase in age is found to raise the probability of being a current drinker[18,21]. As available evidence depicts, drinking is common among middle-aged women,[22,23,24] and elderly women[25,26]. Older age do act as a significant predictor of heavy alcohol use as well[26]. Another possible explanation of late-life drinking may be the response to particular late-life social context and coping mechanisms among women[27].

Education, especially lower level, is a known independent trigger of alcohol consumption[15,28]. Odds of being current drinkers in present study remained significantly lower among secondary and higher education holders, than those of no education group. It holds similarity with findings from other study[28].

Not surprisingly, ethnicity, stood as an important driver of alcohol in present study. The higher prevalence of alcohol consumption in the Jajanati group (previously called Matwali)[9], than other groups across all patterns of alcohol use reflects that group's long-time acceptance of alcohol use in social and cultural life. Socially and culturally diverse Nepal has ambivalent drinking cultures, including sternly negative and prohibitive attitudes towards drinking among e.g. the upper caste[7]. Negative attitudes towards alcohol are also prevalent among the study’s religious minorities (Muslim).

Substantially higher proportion of drinkers from mountain may be accounted to ecology driven socio-cultural environment, reflecting the strong belief that alcohol help people cope extremely cold weather and in line with a study in Bhutan showed a higher prevalence (30%) of women drinker in mountainous area[29]. In contrast, women from the Terai were less likely to drink currently, in the last 12 months or at any time in their life which is in consistent with a recent nationwide WHO STEPS survey on NCD risk factors [30].

In line with previous study[20], MWRAs with a drinking spouse had higher probability of being drinker. An unfortunate link in light of Nepalese men being heavy drinkers and the risk of adult drinking [7], for children growing up in such environment. In addition, alcohol consumption across all the patterns was also significantly higher among those who had home brewing of alcoholic beverages. People brew alcohol at home mainly for cultural reasons with the purpose of self use. The strong association found between home brewing and alcohol consumption behavior predict huge challenges regarding planning of alcohol use reduction interventions.

A well known fact, alcohol increases the chances of NCDs[31]to infectious diseases,[32,33] injuries,[34], and a number of negative social consequences[34]. In the light of rising burden of NCDs in Nepal,[35] assessment of alcohol consumption among this vulnerable populace is justifiable, as it's a matter of equity for both woman and her child.

MWRA’s alcohol consumption can have devastating consequences for the children they give birth to: low birth weight, pre-term birth, small for gestational age,[36] fetal alcohol spectrum disorders,[37] and birth defects[38]. Alcoholic women may also encounter violence[39], depression, and even reproductive health problems,[40] especially if they have heavy drinking behavior.

The study followed a number of scientific approaches to enhance its strength. Notable to mention are: enough sample to assure power of the study, representativeness of study samples, and use of the study instrument developed based on the global standard tools and further validated in the local context.

Nevertheless, this study was not free of limitations as well. Cross-sectional design of the study did not allow explaining the causality. Chances of recall bias, especially unintentional one (especially in alcohol consumption in last 12 months) is too high to ignore. As drinking is regarded as business of men in Nepal[9], possibility of conscious under-reporting (intentional information bias) could not be ruled out as well.

This study is one of the pioneer studies to report alcohol consumption practice and significant correlates among MWRA at the national level. Study's findings as such might be helpful for policymakers and program planners in the context of ongoing efforts to take legal measures for reducing alcohol consumption, and most importantly improving pregnancy outcomes in Nepal.

In conclusion, based on the MWRAs identified to have the highest risk of current alcohol consumption, alcohol-reducing interventions should be targeted especially at MWRAs of Janajati ethnic origin living in mountains, who do not have more than primary education, who have husbands who drink alcohol and live in families that brew alcohol at home. Even though 40−49-year-old women had a higher risk of drinking, one has to consider that younger women are more likely to bear children, and aim interventions at target groups with the best potential for both mother and child.

Taking this study as a reference, future research should focus on assessing and improving pregnancy outcomes among alcohol consuming women within various strata in Nepal in a culturally sensitive way.

## Supporting Information

S1 Codebook Variable codebook final. (XLSX)

S1 Dataset Dataset alcohol women 2014. (XLSX)
